# Can ovaries be preserved after an ovarian arteriovenous disconnection? One case report and a review of surgical treatment using Da Vinci robots for aggressive ovarian fibromatosis

**DOI:** 10.1186/s13048-019-0528-y

**Published:** 2019-06-07

**Authors:** Jun Ying, Jiawen Feng, Jinghui Hu, Shuo Wang, Peilin Han, Yujie Huang, Wei Zhao, Jianhua Qian

**Affiliations:** Department of Gynaecology, The First Affiliated Hospital, ZheJiang University School of Medicine, Zhejiang, Hangzhou China

**Keywords:** Aggressive fibromatosis, Surgery, Ovarian vessels, Ovary protection

## Abstract

**Background:**

The ovary is an important organ of the female reproductive system, which produces oocytes and secretes reproductive hormones. Ovaries have complex dual blood supplies with their blood supply being the core component to protect and ensure ovarian function. Ovarian preservation surgery often encounters problems related to whether or not to preserve ovarian vessels on the affected side.

**Case presentation:**

This study reports on the case of a 30-year-old female patient with the retroperitoneal fibromatosis that had a history of uterine leiomyoma. During the operation, the ovarian arteries and veins were separated according to what was found during the procedure. A postoperative examination demonstrated good function and morphology of the ovary.

**Conclusions:**

A thorough review of academic journals combined with our collection of clinical data was conducted, which confirmed the double blood supply source to the ovaries. As a result of this exploration, a new surgical method is being proposed that is designed to protect the ovaries. By conducting this new procedure, the patient’s disease was not only halted and ultimately cured, but results demonstrate that the method was also able to retain the shape and function of the ovary. The postoperative satisfaction of the patient was significantly improved.

## Background

Considered to be of the most important reproductive organs of women, the ovary has two main functions: producing germ cells and secreting endocrine hormones. It is worth mentioning that ovaries have a complex blood supply system. This is largely due to ovarian arteries and uterine arteries, which are required to support ovarian functions [1]. Common diseases in women often affect the blood supply of ovarian arteries, such as adnexal torsion and pelvic masses. Malignant tumors of the reproductive system are especially common in elderly women [2]. Normally, when an ovarian artery is twisted or invaded by a mass, a surgeon will choose to perform a procedure to rectify the problem and remove the affected ovary at the same time. This study is reporting on a case of aggressive fibromatosis involving an ovarian artery, in order to demonstrate a new and alternative ovarian protection procedure.

Aggressive fibromatosis is a benign tumor composed of differentiated fibroblastic cells with locally aggressive features. However, it does not metastasize [3]. Fibroblastic cells with bands of collagen and an ill-defined cytoplasm rarely show mitosis [4]. It has a low incidence and only accounts for about 0.03% of all tumors. Relevant literature reports that the annual average incidence of the disease in the general population is about 0.002–0.004‰. Most of these incidences are sporadic cases, occurring mainly in women, and especially in patients aged 10–40 years [5, 6]. At present, surgery is the primary method of treatment with the standard surgical goal to completely resect the lesion of negative margins [7, 8]. Aggressive fibromatosis occurring in the posterior peritoneum can invade peripheral blood vessels and nerves. How to deal with the complexities regarding ovarian blood vessels has become a problem worthy of further investigation.

During the course of current procedure method, the invaded blood vessels and the organs they supply are usually removed. However, in this study, in order to completely resect the tumor, a procedure was adopted that cut off the ovarian blood vessels on the affected side to protect the ovary. This is a new and innovative procedure that has been designed to preserve the ovary.

## Case presentation

**Fig. 1:**
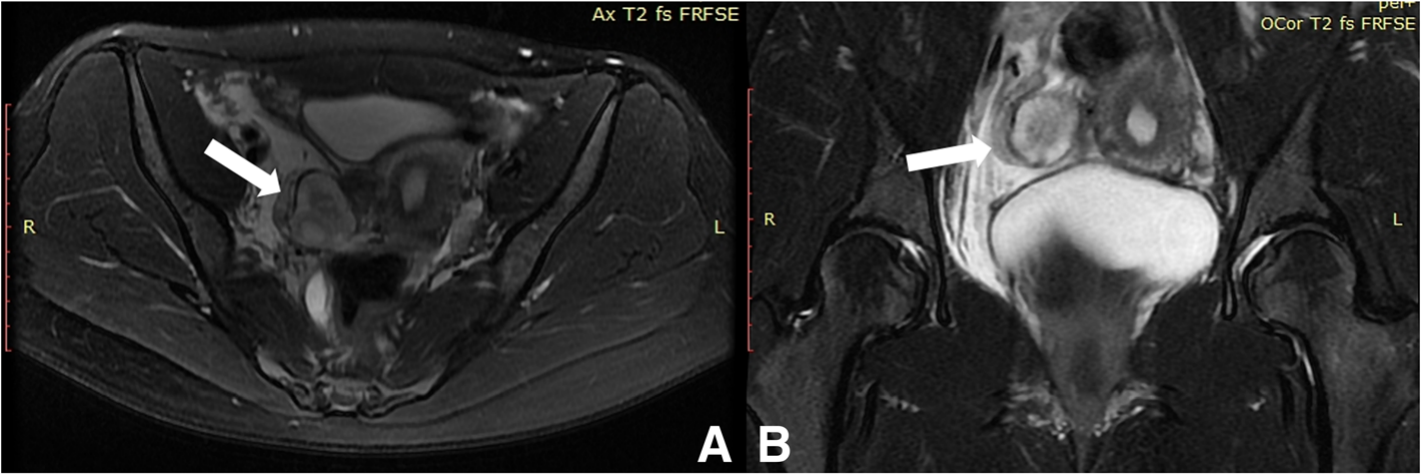
Pelvic magnetic resonance imaging: typical pelvic fibroma (white arrow) presenting as a well-delineated mass on the T2-weighted image. **a** T2-weighted image, transverse section; **b** T2-weighted image, coronal section

**Fig. 2:**
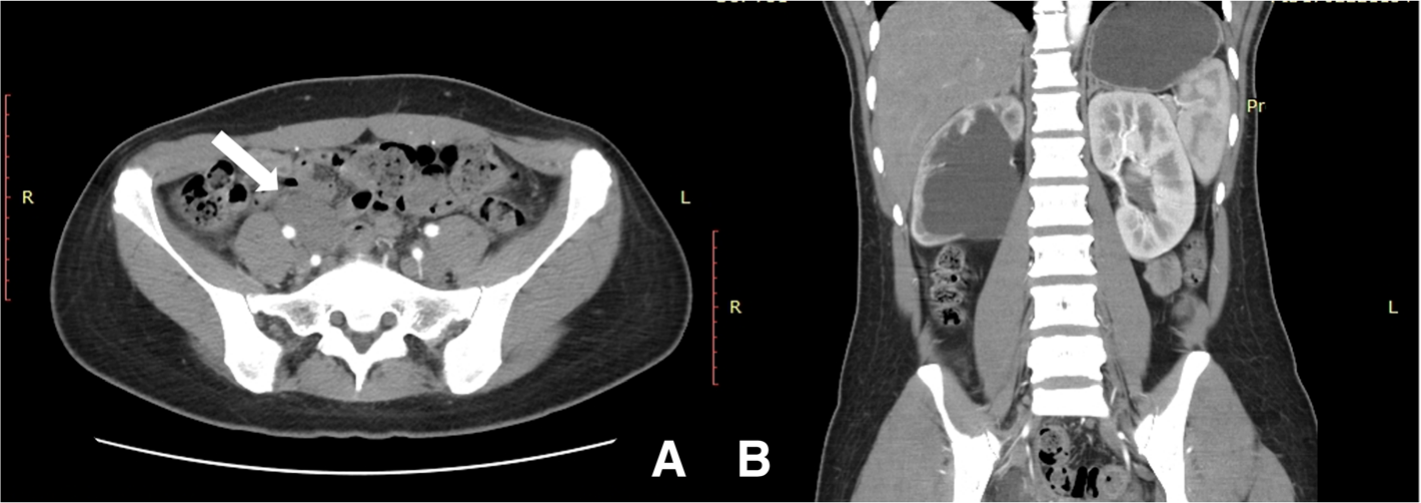
Abdominal CT imageing: pelvic tumors (white arrow) compress right ureter, and cause severe hydronephrosis on the right. **a** transverse section; **b** coronal section

**Fig. 3:**
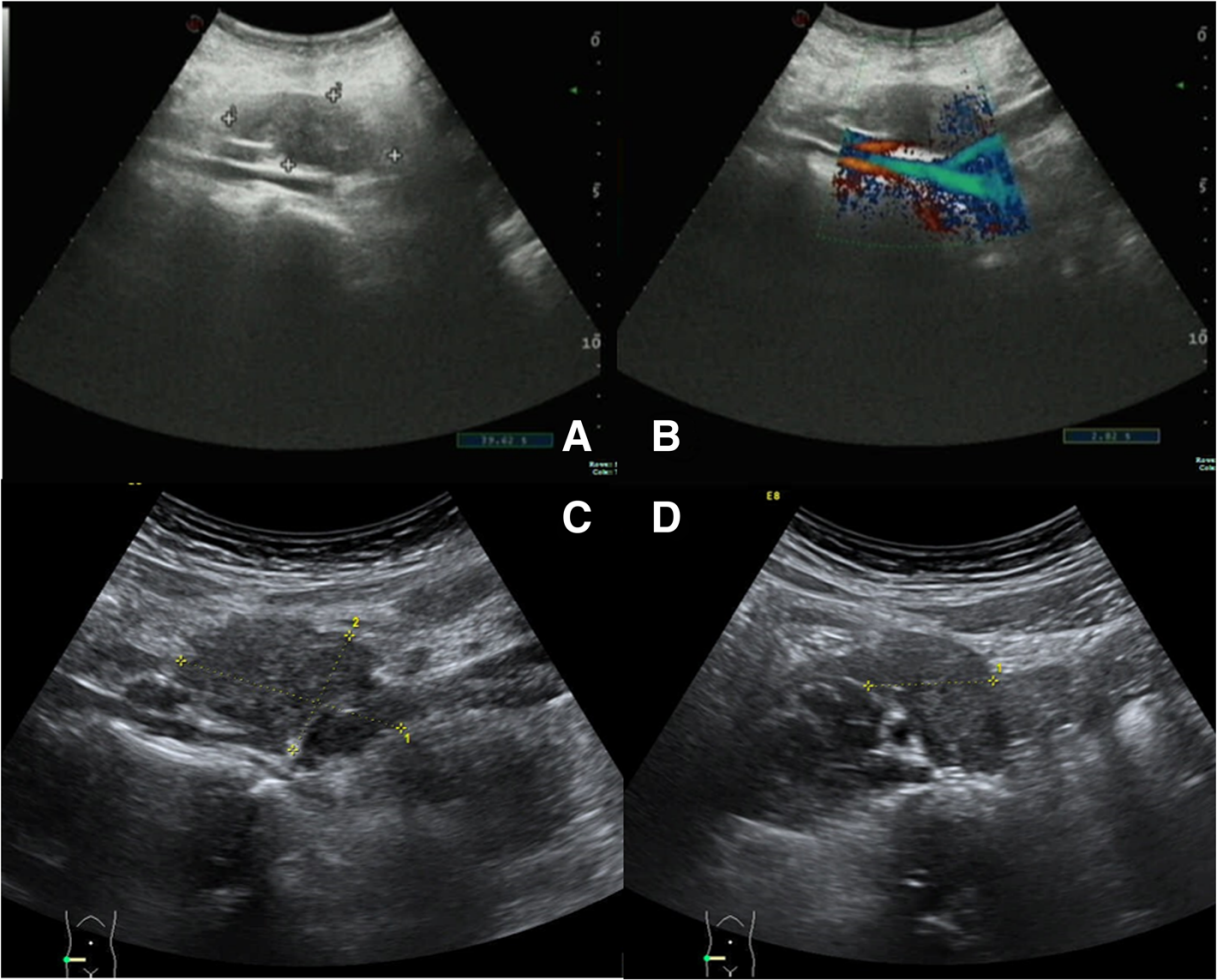
Preoperative imaging. **a** and **b** Color Doppler ultrasound (double kidneys, ureters, bladder); **c** and **d**: Transvaginal color Doppler ultrasonography

A 30-year-old woman visited the hospital due to right lower quadrant pain over the period of 1 week. A laparoscopic myomectomy was performed 4 years ago because of a broad ligament leiomyoma, which was about 10 cm in size. Laboratory findings included a routine blood examination, and a C-Reactive Protein (CRP) test, with tumor markers all found to be within normal ranges. The pelvic Magnetic Resonance Imaging (MRI) scan showed some nodules at the posterior margin of the bladder that were considered to be endometriosis, and some pelvic effusion that was significant on the right side. It was also noted that there was a mass shadow at the lower right ureter (medial to the iliopsoas muscle), with distention of the lower right ureter (Fig. 1). The patient also underwent computed tomography (CT) scans to enable the practitioners to observe the size of the abdominal mass and its surroundings. The CT images revealed a region occupying the middle right ureter that was considered to be a retroperitoneal aggressive fibroma, which led to severe hydronephrosis on the right kidney and upper ureter, and a right pelvic effusion (Fig. 2). After more detailed examinations were conducted, there were no obvious abnormalities detected in the brain, heart, liver, gallbladder, spleen, pancreas or blood. The color Doppler ultrasound demonstrated that there was a hypoechoic mass next to the right iliac vessels that was closely related to the adjacent ureter. This resulted in severe hydronephrosis of the right kidney and a right upper ureteral dilatation (Fig. 3). Ndzengue et al. [7] reported a case of a pelvic desmoid tumor simulating a uterine leiomyoma recurrence. The patient that presented at our hospital had a history of uterine leiomyoma. We subsequently organized a multidisciplinary consultation to determine the next stage of her treatment plan. According to the patient’s surgical history, the next step would be determined after reviewing the results of the last surgical pathological wax, because the pathological nature of the retroperitoneal mass was uncertain.

**Fig. 4:**
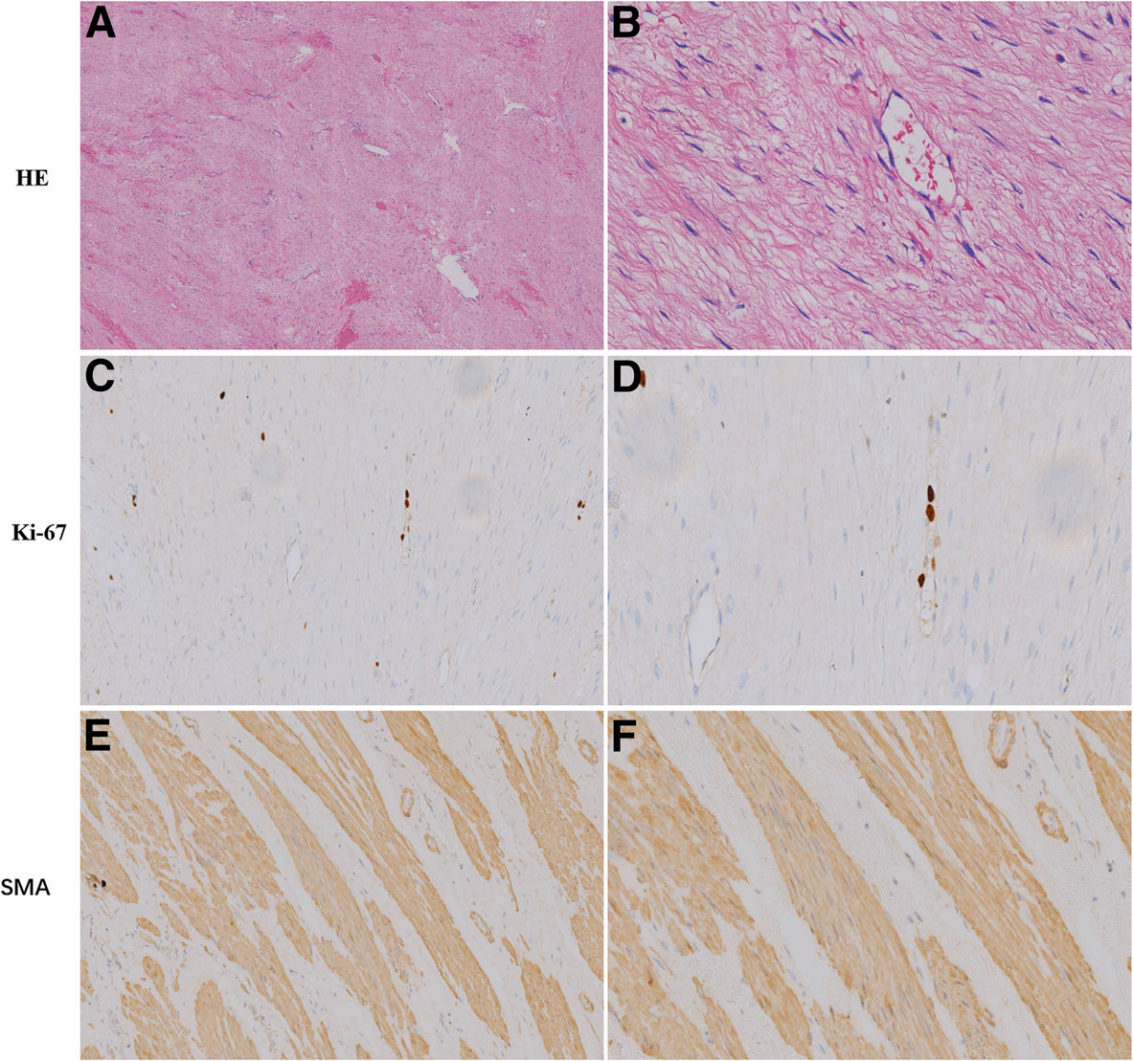
Hematoxylin and eosin (HE) staining shows the tumor cells were arranged sparsely in a spindle shape with blood vessels of different sizes in interstitial tissue. Hematoxylin and eosin [HE]staining: (**a**: 40× magnification; **b**: 400× magnification); Immunostaining for Ki-67: (**c**: 100× magnification; **d**: 200× magnification); Immunostaining for SMA: (**e**: 100× magnification; **f**: 200× magnification)

**Fig. 5:**
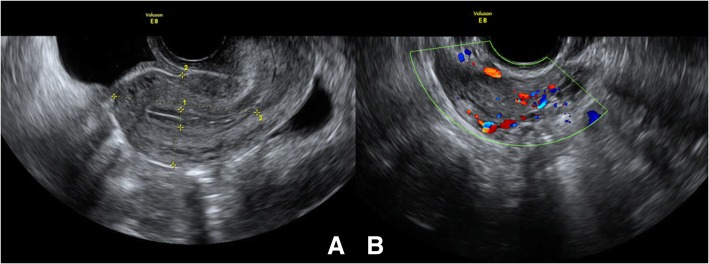
Postoperative images: **a** and **b** Transvaginal color Doppler ultrasonography. **a** uterus, median sagittal section; **b** The right ovary has complete shape and good blood supply

Consequently, a laparoscopic pelvic tumor resection with assistance from a Da Vinci robot was successfully conducted. A local peritoneal protuberance was observed in the right iliac vascular area. The parietal peritoneum was incised above the mass and it was carefully freed along the edge of the mass. The size of the tumor was about 6.0 cm × 5.0 cm × 3.0 cm. It had invasively grown and it was wrapped around the right ureter and the right ovarian arteries and veins. It was stuck to the psoas muscle and the iliac vessels. According to preoperative preparations and intraoperative conditions, a decision was made to cut off the right ureter, the right ovarian arteries and veins, and completely remove the tumor and the two masses that were located in front of the broad ligament on the right hand side of the uterus. The sizes of the masses were approximately 1.5 cm × 1.0 cm × 1.0 cm. The right ureter was anastomosed and put into a double J tube. A pathological diagnosis of an intraoperative frozen sample determined that it was a spindle cell soft tissue tumor, and the two masses were leiomyomas. Postoperative pathology tests of the pelvic mass determined that it was aggressive fibromatosis that had invaded the ureteral wall (Fig. 4). The uterine surface nodules were also leiomyomas. Microscopically, the tumor cells were arranged sparsely in a spindle shape with blood vessels of different sizes found in the interstitial tissue. Immunohistochemical findings were found to be partially positive for smooth muscle actin (SMA) and desmin, and less than 5% ki-67 of positive cells were seen in the lesion. A detailed re-examination was performed 3 months after the initial surgery, to determine the structure and function of the ovaries. The transvaginal color Doppler ultrasonography was able to determine that the ovaries were normal in size with several follicular echoes. The blood supply to the right ovary was good. There were no obvious abnormalities in the uterus or pelvic cavity (Fig. 5). The pelvic MRI and the CT scan of the whole abdomen determined that there were no abnormal lesions in the pelvis. Simultaneously, the endocrine function of the patient’s ovaries was found to be normal, and she was able to self-maturate after removing the double J tube.

## Discussion

According to available literature, aggressive fibromatosis is a rare tumor of monoclonal fibroblastic proliferation, which is characterized by variable and complicated clinical processes [3, 4], These are divided into three categories depending on their anatomical location: extra-abdominal (60%), abdominal wall (25%), and intra-abdominal (8–15%) [9]. The Intra-abdominal AF often occurs in the small intestinal mesentery, but it can also occur in the posterior peritoneum, ileocolic mesentery, gastrocolic ligament and greater omentum. The diagnosis can be confirmed by additional examinations, such as an MRI, which is the method of choice when fibromatosis and recurrence are suspected [10], as well as pathology and immunohistochemistry [11, 12]. Presently, surgery is the main and most effective method of treatment, with the standard surgical goal to completely excise the lesion with negative surgical margins [7, 8]. The postoperative recurrence rate is 13–68% and the recurrence generally occurs more than 2 years after the initial surgery [13]. Multiple recurrences may lead to a wider range of lesions, and even endangering life further by its invasion of important organs. It is also likely that the recurrence and mortality rate after a second operation will also increase significantly. A retrospective, multicenter analysis showed that the effect of the laparoscopic surgery (LS) and a robot-assisted laparoscopic (RALS) approach for the treatment of early-stage ovarian cancer is comparable to existing treatments [14] To fully expose the anatomical structure of the lesion, a RALS approach was selected. In this study, the mass was completely removed, and the margin of incision was negative, which reached the required standards for operating procedures. There were no recurrences detected in the 18 months after the operation, and all of the patient’s organs affected by the tumors were able to demonstrate good morphology and function.

It is worth noting that this mass invaded the right infundibulopelvic ligament and the right ovary arteries and veins. The right ovary’s arteries and veins were removed during the operation. However, the function and morphology of the right ovary was not significantly or negatively affected after the operation. This led the researchers to further explore the anatomical structure of ovarian arteries and veins, as well as its blood supply system. Relevant anatomical data has reported that ovaries have a double blood supply system, which comes from the ovarian artery (OA) and the ovarian branch of the uterine artery [1]. The origin of the ovarian artery is relatively constant and regular. A total of 95.8% of the OA originates from the anterolateral part of the abdominal aorta. The uterine artery originates from the branch of the anterior trunk of the internal iliac artery [15]. There are four known models related to the ovarian blood supply. Type I: the main ovarian artery is anastomosed with the ovarian branch of the uterine artery at the ovarian hilum, and both are supplied with blood equally, which accounts for 72.5%. Type II: the main ovarian artery and the ovarian branch of the uterine artery form a loop shape, and both are supplied with blood equally, which accounts for 13.7%. Type III: the ovarian branch of the uterine artery is anastomosed with a small branch of the ovarian artery, with the blood supply of the ovary mainly supplied by the uterine artery, which accounts for 10%. Type IV: the ovarian artery and the uterine artery are anastomosed at the uterine end of the fallopian tube, and the blood supply of the ovary is mainly supplied by the ovarian artery, which accounts for 3.7% [15]. Such statistics demonstrate that only 3.7% of cases rely mainly on ovarian arteries for their blood supply. This provides a theoretical basis for the surgical approach proposed in this study, with the primary objective to preserve and avoid the removal of the ovaries.

This theory of ovarian vascular anatomy can also be applied to the treatment of another disease. Ovarian tumor pedicle torsion is a very common gynecological acute abdomen. Over recent years, a new surgical method of ovarian preservation has been developed. A ligation of the ovarian arteries and veins is conducted, but not cut off, with the reversed appendages repositioned and the ovarian cysts removed [16]. This approach not only retains the morphology and function of the ovary, but it also blocks the channel of thrombus shedding, making the thrombus remain in the ovary vessels, and it avoids the occurrence of a pulmonary artery embolism. It also blocks the blood supply from the ovarian arteries, with the uterine artery and collateral vessels needed for a continued blood supply. This type of ovarian tumor generates a lot of collateral circulation before the procedure, which is a certain source of blood supply for ovaries. Therefore, several months after the initial procedure and during a follow-up examination, the appearance and function of the patient’s ovaries were found to be postive. Huang et al. [17] analyzed the surgical method applied during 42 cases of ovarian cyst pedicle torsion. Their findings determined that when compared with traditional surgical methods of ovarian resection, ovarian preservation surgical methods have distinct advantages for postoperative endocrine function recovery, and it has additional safety and effectiveness outcomes [17]. Taskin et al. [18] established the mouse model with uterine adnexal torsion in 1998. They found that the ovary arteriovenous occlusion in mice caused ischemia for 12–24 h. Even after 36 h, and although the ovaries were a blue-black color, the retained ovaries still had normal functions. This outcome demonstrated the possibility of the ovary’s survival even after cutting off the ovarian arteries and veins, from the perspective of histology and biochemistry [18]. However, Lagana et al. [19] emphasized a pathological process to maintain the circulation of the ovary after detorsion, deteriorates the tissue injury and leads to a pathologic process called ischemia/reperfusion (I/R) injury. This is characterized by oxidative stress. It is suggested that researchers should develop shared protocols for clinical use and application [19]. Sipahi et al. [20] conducted a controlled trial to demonstrate that remote ischemic modulation (RIC) can alleviate ovarian ischemia/reperfusion injuries in rats. These studies have contributed to laying the theoretical foundation for the success of this proposed procedure.

## Conclusion

A thorough exploration of existing literature and an in-depth analysis of this case, has confirmed the existence of a double blood supply to the ovaries. In this study, a new surgical procedure was proposed to treat aggressive ovarian fibromatosis. The procedure proposes that when a pelvic tumor invades the ovarian arteries and veins, the ovarian arteries and veins are cut and divided, in order to retain ovarian function. Due to the unique blood supply system of the ovaries and special conditions related to the disease, the procedure that formed the basis of this study, was able to successfully remove the tumors on the premise of preserving the ovaries. Such a ground-breaking surgical method needs to be confirmed for a wider application based on research and shared protocols, indicators, contradictions and specific conditions related to the patient. Only then will practitioners have the confidence to determine whether the procedure is effective and feasible.

## Data Availability

Not applicable.
